# Commitment of Satellite Cells Expressing the Calcium Channel **α**2**δ**1 Subunit to the Muscle Lineage

**DOI:** 10.1155/2012/460842

**Published:** 2012-11-29

**Authors:** Tammy Tamayo, Liliana Grajales, Jesús García

**Affiliations:** Department of Physiology and Biophysics and Center for Cardiovascular Research, University of Illinois at Chicago, 835 South Wolcott Avenue, Chicago, IL 60612, USA

## Abstract

Satellite cells can maintain or repair muscle because they possess stem cell properties, making them a valuable option for cell therapy. However, cell transplants into skeletal muscle of patients with muscular dystrophy are limited by donor cell attachment, migration, and survival in the host tissue. Cells used for therapy are selected based on specific markers present in the plasma membrane. Although many markers have been identified, there is a need to find a marker that is expressed at different states in satellite cells, activated, quiescent, or differentiated cell. Furthermore, the marker has to be present in human tissue. Recently we reported that the plasma membrane **α**2**δ**1 protein is involved in cell attachment and migration in myoblasts. The **α**2**δ**1 subunit forms a part of the L-type voltage-dependent calcium channel in adult skeletal muscle. We found that the **α**2**δ**1 subunit is expressed in the majority of newly isolated satellite cells and that it appears earlier than the **α**1 subunits and at higher levels than the **β** or **γ** subunits. We also found that those cells that expressed **α**2**δ**1 would differentiate into muscle cells. This evidence indicates that the **α**2**δ**1 may be used as a marker of satellite cells that will differentiate into muscle.

## 1. Introduction

 Satellite cells are found between the plasma membrane of the muscle fiber and the basal lamina [1]. They are responsible for the growth, maintenance, and repair of skeletal muscle. They remain in a mitotically quiescent state under normal physiological conditions but can be activated during exercise or muscle damage, aiding with the repair of muscle. Satellite cells can maintain or repair muscle because they possess stem cell properties; they can differentiate into other cell types [2] and can also divide and maintain their population. Activated satellite cells undergo several rounds of cell division, and some of them will differentiate and fuse to form the typical multinucleated skeletal muscle fiber. Due to the regenerative capacities provided by satellite cells, they are a valuable option for cell therapy. Cells used for therapy are selected based on specific markers present in the plasma membrane. In the case of satellite cells the function of those markers ranges from the regulation of proliferation to cell-cycle entry to fusion (reviewed in [3]). Although many markers have been identified, there is a need to find a marker indicative of the cells that will commit to the muscle lineage and that is expressed at different states, that is, activated, quiescent, or differentiated cell. Furthermore, the marker has to be present in human tissue. Here we examined the *α*2*δ*1 protein, a subunit of calcium channels that fulfills those requirements.

 The *α*2*δ*1 subunit forms a part of the L-type voltage-dependent calcium channel (or dihydropyridine receptor, DHPR) in adult skeletal muscle. In addition to *α*2*δ*1, the skeletal muscle DHPR contains *α*1 (Ca_v_1.1), *β*, and *γ* subunits. The *α*1 subunit is the voltage sensor and contains the channel pore [4], while the role commonly assigned to the other subunits is to regulate the activity of *α*1. However, recent evidence has shown that the *β* and the *α*2*δ*1 subunits have roles independent of calcium channels. The *β* subunit is an intracellular protein involved in the regulation of gene expression in different cell types including myoblasts [5–7]. The *α*2*δ*1 protein is an extracellular protein involved in cell attachment and migration and possibly cell signaling in myoblasts [8, 9]. We recently found that the *α*2*δ*1 subunit localizes at the leading ends of myotubes with little or no association with *α*1 subunits 2 days after the induction of differentiation [8] suggesting that, in addition to attachment and migration, *α*2*δ*1 may play a role in the elongation process of myotubes. With longer times in differentiation medium, the localization of *α*2*δ*1 gradually becomes homogeneous until it colocalizes almost completely with *α*1. However, some *α*2*δ*1 subunit does not colocalize with *α*1 even at later times in a number of myotubes. Interestingly, experiments performed in dysgenic muscle (which lack the *α*1 subunit) have shown that the *α*2*δ*1 subunit is normally expressed but that its distribution patterns are abnormal in the absence of *α*1 [10]. In dysgenic muscle cells, *α*2*δ*1 was found in the plasma membrane, around the nucleus, and in the transverse tubular membrane in a diffuse pattern. Accordingly, the localization pattern of *α*2*δ*1 in dysgenic cells closely resembles our findings in immature muscle where there is little or no *α*1 subunit to associate with *α*2*δ*1. These data indicate that the *α*2*δ*1 subunit is not only part of the DHPR but that it may be important for other cellular functions in muscle precursor cells or satellite cells. Thus, the purpose of the present study was to determine whether the *α*2*δ*1 subunit is present in satellite cells and, if so, when the *α*2*δ*1 subunit first appears in those cells. We also sought to determine the fate of satellite cells expressing *α*2*δ*1. We found that the *α*2*δ*1 subunit is expressed in the majority of newly isolated satellite cells and that it appears earlier than the *α*1 subunits and at higher levels than the *β* or *γ* subunits. We also found that those cells that expressed *α*2*δ*1 would differentiate into muscle cells. This evidence indicates that the *α*2*δ*1 may be used as a marker of satellite cells that will differentiate into muscle.

## 2. Methods

### 2.1. Isolation of Satellite Cells

All experiments using animals were approved by the Institutional Animal Care and Use Committee at the University of Illinois at Chicago. Skeletal muscle of newborn mice (<72 hours postnatal) was dissociated for fluorescence-activated cell sorting (FACS) and used for the extraction of total RNA (see below). Dissociation of muscle was performed in Ca^2+^-, Mg^2+^-free Rodent Ringer (in mM): 155 NaCl, 5 KCl, 11 glucose, 10 HEPES, pH 7.4 containing, 0.3% trypsin type XI, 0.01% DNAse I, and 1 mg/mL collagenase type IA (Sigma), as previously reported [11].

### 2.2. Fluorescence-Activated Cell Sorting

 Cells were suspended in sorting media (phosphate-buffered saline (PBS) with 10 mM HEPES and 0.5% BSA) and labeled with antibodies against CD34 (anti-mouse CD34 conjugated with Alexa Fluor 700; eBiosciences) and *α*2*δ*1 (monoclonal antibody 20 A from Pierce Thermo Scientific and a secondary pac blue goat anti-mouse antibody from Invitrogen). Collection tubes contained growth media (high-glucose DMEM supplemented with L-glutamine, 10% equine serum, 10% fetal bovine serum, and 1% penicillin/streptomycin). Immediately after the sort was completed, the cells were centrifuged, resuspended in Dulbecco's modified essential medium supplemented with 4.5 g/L glucose, 10% horse serum, and 10% fetal calf serum, and plated at a density of 26,000 cells per square centimeter on 35 mm primaria dishes.

### 2.3. Real-Time Quantitative Polymerase Chain Reaction (RT-qPCR)

Total RNA was obtained from satellite cells from two 35 mm culture plates for each day (0–7). RNA extractions were done following Qiagen RNeasy mini-kit followed by DNAse treatment to avoid amplification of genomic DNA. The RNA density for each sample was measured (Thermo Scientific: spectrophotometer NanoDrop 8000) and normalized to the lowest RNA density found in the sample group and reverse transcribed to cDNA using ImProm-II kit from Promega with a random primer. RT-qPCR was performed (Applied Biosystems 7500) using 10 *μ*L of Fast SYBR Green Master Mix, 7 *μ*L of molecular grade water, 1 *μ*L of forward, and 1 *μ*L of reverse gene specific primer, plus 1 *μ*L of cDNA. All genes except 18S were run between 59 and 60°C annealing temperature, and dissociation curves were obtained for all gene/cDNA mixes and for the primer without cDNA for control and comparison. The 18S gene was run at 55°C. The number of independent cell cultures analyzed was a minimum of 3, and the same gene/cDNA mix was analyzed 3 times in qPCR 96 well plates. All genes were referenced to the geometric mean of at least two control genes selected from YWHAZ, 18S, and HPRT1 [12, 13]. The primer sequences are given in Table 1.

### 2.4. Statistics Data

Are expressed as means ± SEM. To determine statistical significance, we used one-way ANOVA followed by Tukey's multiple comparison test or two-way ANOVA followed by Dunn's multiple comparison test. A value of *P* < 0.05 was considered to be significant.

## 3. Results

### 3.1. Presence of *α*2*δ*1 Protein in Isolated Satellite Cells

**Figure 1:**
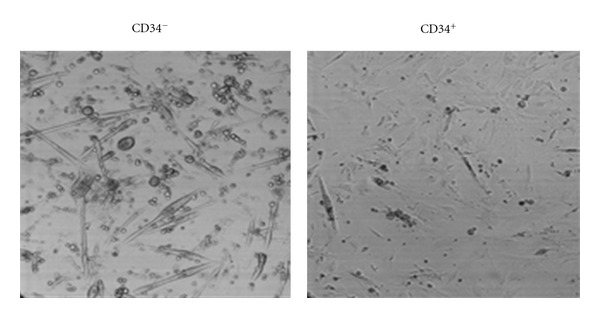
Satellite cells sorted for CD34. CD34^−^ (left) and CD34^+^ (right) cells were plated in growth medium (20% serum). CD34^−^ fused to form myotubes while CD34^+^ remained mononucleated after 7 days in culture. Images are 623 *μ*m × 623 *μ*m.

**Figure 2:**
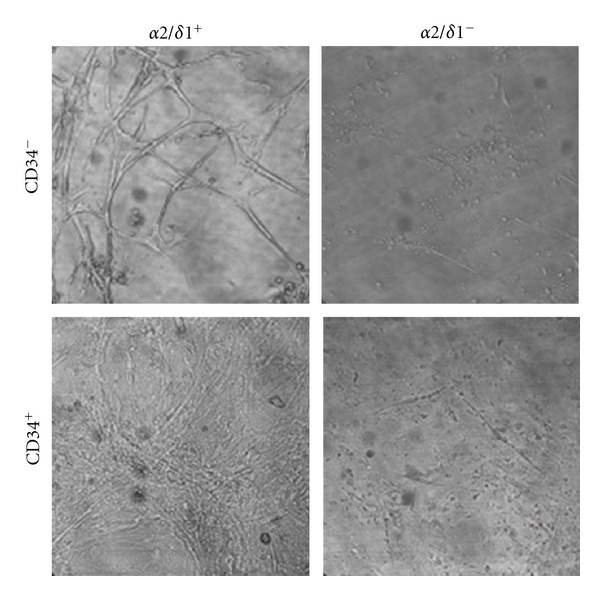
Satellite cells sorted for CD34 and *α*2*δ*1. Satellite cells were sorted in four subpopulations and plated in growth medium (20%). Although both subpopulations of cells expressing *α*2/*δ*1^+^ differentiated into myotubes, the *α*2*δ*1^+^/CD34^−^ cells formed longer and thicker myotubes at 7 and 21 days. *α*2*δ*1^−^/CD34^−^ cells transiently produced myotubes, but they did not last past 10 days in culture. *α*2*δ*1^−^/CD34^+^ cells did not produce myotubes at any time. Images correspond to day 21 in culture. Image sizes are 623 *μ*m × 623 *μ*m.

 Satellite cells were isolated from hind limb muscles from newborn mice and separated by FACS for the presence of the accepted marker CD34 [14]. CD34^+^ and CD34^−^ cells were plated separately on primaria culture dishes and allowed to grow. CD34^−^ cells divided, fused, and developed into myotubes, while most of the CD34^+^ cells remained mononucleated after 7 days in culture, as shown in Figure 1. Differentiation of CD34^−^ cells into myotubes is consistent with other current studies showing that CD34 is lost after satellite cells are activated and that CD34^+^ cells experience little division and remain in a quiescent state [15]. We then performed a double-labeled sort and examined freshly isolated cells for the presence of *α*2*δ*1 since we had previously reported that this protein is expressed early in development of muscle cells and that it is involved in attachment and migration [8, 16]. We found that more than 50% of satellite cells expressed *α*2*δ*1 protein upon isolation, and the majority of these *α*2*δ*1^+^ cells were CD34^+^, as shown in Table 2. The four groups were plated separately in growth media (DMEM, 10% horse serum, 10% fetal bovine serum) and examined at different times in culture. Cells expressing *α*2*δ*1^+^ (CD34^−^) produced myotubes early (2-3 days) and persisted for longer times in culture (>21 days) than the other three groups (Figure 2). Cells *α*2*δ*1^+^/CD34^+^ produced myotubes and also fibroblast-like cells; these myotubes did not last as long in culture as the ones produced in the absence of CD34. Cells *α*2*δ*1^−^/CD34^+^ produced mostly fibroblast-like cells. The *α*2*δ*1^−^/CD34^−^ cells produced myotubes at later times (>5 days) and did not last more than a few days in culture. Cells that were *α*2*δ*1^−^/CD34^−^ at the time of the sort were then examined for the presence of *α*2*δ*1 with RT-PCR. It was observed that *α*2*δ*1^−^/CD34^−^ cells started expressing *α*2*δ*1 after plating, and that they expressed *α*2*δ*1 only during the time when the cells were differentiating into myotubes. After 7 days in culture the level of expression of *α*2*δ*1 in *α*2*δ*1^−^/CD34^−^ cells was 1.82 ± 0.07 (normalized to 18S). In *α*2*δ*1^+^/CD34^−^ and *α*2*δ*1^+^/CD34^+^ the level of expression of *α*2*δ*1 was 0.81 ± 0.03 and 1.01 ± 0.05, respectively, (*n* = 6). These data further support the idea that *α*2*δ*1 is linked to the differentiation of the cells.

### 3.2. The *α*2*δ*1 Protein is Expressed Earlier Than the Other Calcium Channel Subunits

**Figure 3:**
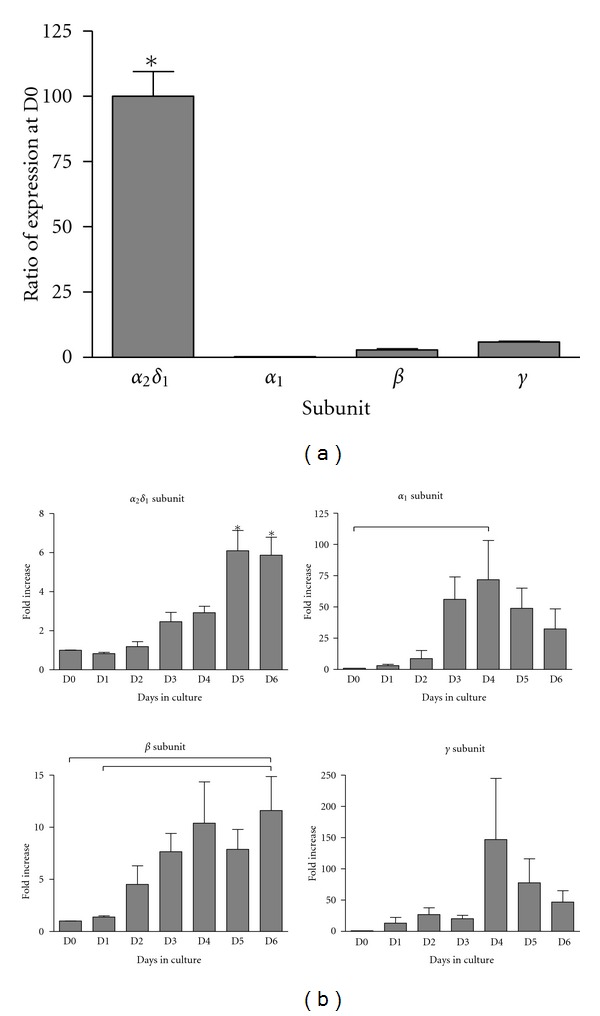
Expression of calcium channel subunits in satellite cells by RT-qPCR. (a) The *α*2*δ*1 subunit is expressed at significantly higher levels than the *α*1, *β*, and *γ* subunits in freshly isolated unsorted satellite cells. Expression of *α*1, *β*, and *γ* subunits was normalized to *α*2*δ*1 levels for comparison. The asterisk indicates statistically significant difference with all other data points (*P* < 0.001). (b) Temporal expression of calcium channel subunits in satellite cells cultured for different times in 20% serum. For each subunit, the fold change was calculated with their respective level at D0. The *α*2*δ*1 subunit showed a smaller fold change with time in culture when compared to the other three subunits, followed by the *β* subunit. The *α*1 and the *γ* subunits showed a large fold change of expression. Bars represent mean ± sem of three independent cultures measured in triplicate in the qPCR plate. Differences between data points are represented by lines. The asterisk represents differences with all other bars except those ones with an asterisk. For all graphs, the *P* value was <0.05. All genes were referenced to the geometric mean of at least two control genes selected from YWHAZ, 18S, and HPRT1.

 We further characterized the temporal expression of *α*2*δ*1 in the total satellite cell population at different times after isolation and compared it with the expression of *α*1, *β* and *γ* subunits by RT-qPCR. The message for the *α*2*δ*1 subunit was present in freshly isolated satellite cells (D0) while the message for the other subunits was very low. To provide an objective comparison among the levels of expression of the different calcium channel subunits, Figure 3(a) shows the ratios of expression of *α*1, *β*, and *γ* subunits in relation to the expression of *α*2*δ*1 at D0. All values were normalized to the expression of *α*2*δ*1 since this subunit had the highest level of expression. The ratios of expression were 0.26 ± 0.04%, 2.89 ± 0.38%, and 5.89 ± 0.34% for *α*1, *β*, and *γ* subunits, respectively. These values were significantly lower than those of *α*2/*δ*1 at D0 (*P* < 0.001).

 Because the levels of expression of the subunits were so different at D0, it was expected that the expression for each subunit would not increase proportionately with differentiation. For the *α*2*δ*1 subunit its levels were significantly higher (6-fold) by D5 compared to D0 and remained high by D6 (Figure 3(b)). In contrast, levels of *α*1 were barely detected at D0. Low levels of *α*1 were detected by D1 but increased significantly (~70-fold) by D4 and stayed around this level through D6. Similarly to *α*1, the levels of *β* subunit increased more than 10-fold by D4 compared to D0. The *γ* subunit levels increased with time to reach a maximum 147-fold increase at D4 compared to D0. Thus, the fold increase in the levels of *α*1, *β*, and *γ* were substantially higher than for *α*2*δ*1. Overall the mRNA data demonstrate that *α*2*δ*1 is expressed earlier than *α*1 and at higher levels than *β* or *γ* subunits in satellite cells and are consistent with studies detecting different protein levels of a2*δ*1 and *α*1 subunits early in development [9, 17].

### 3.3. Quantification of Cell Size

**Figure 4:**
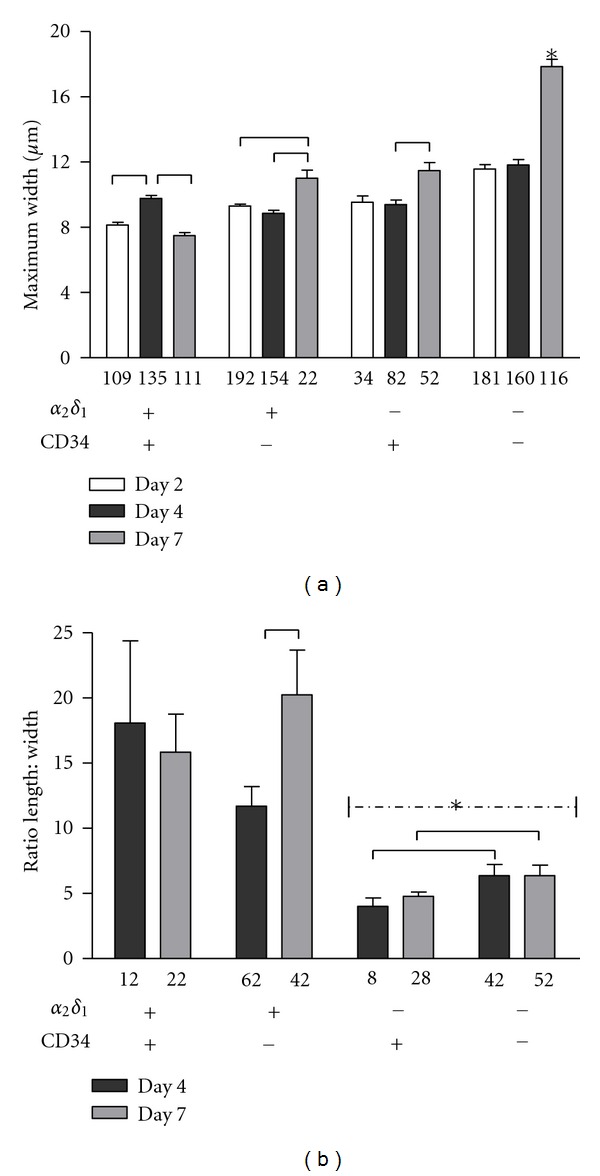
Morphological dimensions of sorted satellite cells. (a) Maximum width was recorded from several random fields for each subpopulation of cells, at 2, 4, and 7 days after culture in 20% serum. The number of cells measured for each subpopulation at each day is indicated below each bar. Statistical differences between data points are represented by lines (*P* < 0.05). The asterisk indicates statistically significant difference with all other data points (*P* < 0.001). (b) Aspect ratio length : width measured from the four subpopulations at days 4 and 7 in culture. The number of cells for each condition is indicated below each bar. The populations expressing *α*2/*δ*1^+^ at the time of sorting had the largest aspect ratios of the four groups at day 4, and the ratio was even larger for the *α*2*δ*1^+^/CD34^−^ group at day 7. Statistical differences between data points are represented by lines (*P* < 0.01). The discontinuous line with an asterisk represents statistically significant difference between *α*2*δ*1^−^ cells and *α*2*δ*1^+^ cells (*P* < 0.001).

 In order to obtain an objective aspect of cell morphology and development of each of the four subpopulations of sorted cells, we measured cell dimensions at several days after initial plating. To facilitate the measurement of differences over time, cells were plated and maintained in the initial growth media. Wide-field images were recorded from several random places in culture dishes. The maximum width and length were measured with Image J software. The maximum width varied between 8 and 12 *μ*m for all cell groups at days 2 and 4 after plating. After 7 days in culture, the width increased in the *α*2*δ*1^+^/CD34^−^, *α*2*δ*1^−^/CD34^+^, and *α*2*δ*1^−^/CD34^−^ groups, with the increase being more significant in the latter (*P* < 0.001). The width showed a small decline in the *α*2*δ*1^+^/CD34^+^ group (Figure 4(a)). Since myoblasts align end to end to form the typical myotubes, we measured the length of the cells and estimated its relation to cell width. The aspect ratio length : width, a measure of elongation, is shown in Figure 4(b) for the four subpopulations at 4 and 7 days. A ratio of 1 would represent a spherical cell. The ratio at day 2 was close to 1 in all groups and is not plotted in the graph. The populations expressing *α*2/*δ*1 at the time of sorting had the largest aspect ratios of the four groups at day 4 (*α*2*δ*1^+^/CD34^+^, 18.1 ± 6.3; *α*2*δ*1^+^/CD34^−^, 11.7 ± 1.5; *α*2*δ*1^−^/CD34^+^, 4 ± 0.7; *α*2*δ*1^−^/CD34^−^, 6.4 ± 0.8) and the ratio was even larger for the *α*2*δ*1^+^/CD34^−^ group at day 7 (*α*2*δ*1^+^/CD34^+^, 15.8 ± 2.9; *α*2*δ*1^+^/CD34^−^, 20.2 ± 3.4; *α*2*δ*1^−^/CD34^+^, 4.8 ± 0.3; *α*2*δ*1^−^/CD34^−^, 6.4 ± 0.8). These measurements are consistent with the idea that cells expressing *α*2*δ*1 form myotubes, as noted above. By day 7 few *α*2*δ*1^−^/CD34^−^ cells had formed myotubes. Instead, cells of this population were compact and multinucleated in appearance. The results are also consistent with the appearance of *α*2/*δ*1 at the leading edges of immature muscle cells [8].

### 3.4. Detection of Myogenic Regulatory Factors

**Figure 5:**
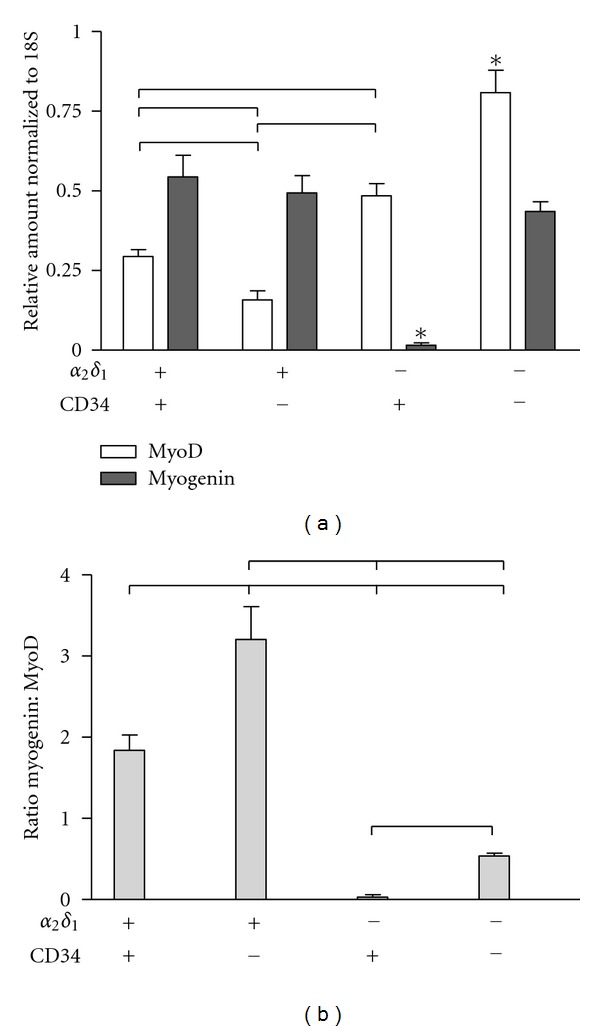
Expression of myogenic transcription factors in satellite cells. (a) Expression levels of MyoD and myogenin were measured in the four subpopulations after two days in culture. Cells expressing *α*2*δ*1^+^ had significantly larger expression of myogenin than MyoD, while *α*2*δ*1^−^ cells expressed larger levels of MyoD than myogenin. Statistical differences between data points are represented by lines (*P* < 0.01). The asterisks represent significant differences of that value with respect to all other data points. (b) The ratio of myogenin to MyoD was significantly higher in *α*2*δ*1^+^-expressing cells, indicating a more advanced differentiation into myotubes than *α*2*δ*1^−^ cells. *P* values ranged from <0.01 to <0.001.

To determine further the differentiation state of the sorted satellite cells and the relationship with *α*2*δ*1 expression, total RNA was isolated from each of the four groups of cells two days after plating in growth media to measure the levels of the myogenic regulatory factors MyoD and myogenin. The values were normalized to the expression of 18S (Figure 5(a)). The presence of the two regulatory factors was detected in the four groups although in different proportions. Cells expressing *α*2*δ*1 had significantly larger expression of myogenin than MyoD (*α*2*δ*1^+^/CD34^+^, MyoD 0.29 ± 0.02, myogenin 0.54 ± 0.07, *P* < 0.01; *α*2*δ*1^+^/CD34^−^, MyoD 0.16 ± 0.03, myogenin 0.49 ± 0.05, *P* < 0.01). The difference in MyoD expression between the *α*2*δ*1^+^-expressing cells was also significant (*P* < 0.05). Cells without expression of *α*2*δ*1 at the time of the sort had a larger amount of MyoD message than myogenin (*α*2*δ*1^−^/CD34^+^, MyoD 0.48 ± 0.04, myogenin 0.02 ± 0.01, *P* < 0.01; *α*2*δ*1^−^/CD34^−^, MyoD 0.81 ± 0.07, myogenin 0.44 ± 0.03, *P* < 0.01). However, as mentioned above, *α*2*δ*1^−^/CD34^−^ started expressing *α*2*δ*1 after plating and thus the differentiation of these cells into muscle cells was delayed in comparison to the cells that expressed *α*2*δ*1 during sorting. Levels of MyoD were also significantly different between the *α*2*δ*1^−^ cells (*P* < 0.001). In contrast, the levels of myogenin were significantly different only in the *α*2*δ*1^−^/CD34^+^ population compared to the other three subgroups (*P* < 0.001). The presence of MyoD and low myogenin is indicative of a population of proliferating cells, while high myogenin and low MyoD, and suggests that these cells are in the process of differentiation [18]. The relationship between these two myogenic regulatory factors can be better appreciated by the ratio of myogenin to MyoD shown in Figure 5(b). Cells expressing *α*2*δ*1 had significantly larger ratios than *α*2*δ*1^−^ cells suggesting that *α*2*δ*1^+^ cells are committed to the muscle lineage. These results agree with the different capacity of the cells in each of the four groups to form myotubes as described above and further support the idea that the expression of the *α*2*δ*1 subunit is a good indicator of the fate of satellite cells.

## 4. Discussion

 Satellite cells are naturally involved in the maintenance and repair of skeletal muscle. This function of satellite cells is of paramount significance in patients with muscular dystrophy. In order to improve the repair of dystrophic muscle, it is necessary to supply muscle with an adequate number of satellite cells with an intact copy of the affected gene. Thus, it is evident that the population of satellite cells used for therapy needs to be enriched with a marker that is indicative of the commitment of the cells to become muscle. Here we have shown that satellite cells that express the *α*2*δ*1 protein are more likely to differentiate into muscle cells *in vitro*. 

 Satellite cells that expressed *α*2*δ*1 at the time of sorting were more likely to differentiate into muscle cells than the cells devoid of this protein. Some of the *α*2*δ*1^−^/CD34^−^ cells, however, also differentiated into muscle cells but at a much later time than cells expressing *α*2*δ*1. The *α*2*δ*1^−^/CD34^−^ cells expressed a2*δ*1 after they had been in culture for several days, effectively turning into *α*2*δ*1^+^/CD34^−^ cells; the absence of CD34 alone did not seem to be enough for the cells to commit to the muscle lineage at early times. This result suggests that it may be optimal to induce the expression of *α*2*δ*1 prior to cell sorting in order to obtain a larger population of cells that will differentiate into skeletal muscle.

 Commitment of cells to the muscle lineage in the presence of *α*2*δ*1 was also demonstrated by the expression of the myogenic regulatory factors MyoD and myogenin two days after plating. *α*2*δ*1-expressing cells at time of sorting expressed relatively higher levels of myogenin than MyoD. In contrast, the levels of MyoD were higher than those of myogenin in cells without *α*2*δ*1 at time of sorting. This suggests that cells expressing *α*2*δ*1 are in a more advanced state of commitment toward muscle than cells without *α*2*δ*1.

 Further support for our proposing a role for the *α*2*δ*1 subunit as a marker of muscle commitment of satellite cells is provided by the fact that *α*2*δ*1 appears earlier than the *α*1, *β*, and *γ* subunits and its levels remain high through the differentiation process. This is the first paper that examines the expression of all the calcium channel subunit in the same cell type. These results also indicate that the assembly of the skeletal muscle DHPR as a complex occurs at later times in development than it was previously believed [19, 20] since not all the subunits are expressed initially and simultaneously.

 An important property of cells expressing *α*2*δ*1 is that they exhibit improved adhesion and migration *in vitro* compared to cells without *α*2*δ*1, as we previously demonstrated [8]. Interestingly, this capability imparted to the cells by the *α*2*δ*1 is not limited to muscle cells and has been confirmed by another laboratory using bone cells and bone matrix (Thompson & Farach-Carson, personal communication). Involvement of *α*2*δ*1 in attachment and migration is a significant attribute for the selection of this protein as a marker of satellite cells since few satellite cells have been shown to migrate great distances from the site of injection in dystrophic muscle. Further experiments must be performed to test the ability of *α*2*δ*1-expressing satellite cells to attach and migrate away from the site of delivery in dystrophic muscle. We previously observed that *α*2*δ*1 is found on the leading edges of cells, and here we show that cells that exhibit early expression of *α*2*δ*1 elongate faster than cells without *α*2*δ*1. In addition to migration and attachment, *α*2*δ*1 may be actively involved in elongation of myotubes. An additional advantage of the *α*2*δ*1 protein is that it is also present in human cells. This fact makes *α*2*δ*1 an ideal candidate to use when isolating cells because many of the other markers present in mouse cells have not been characterized in human cells, and therefore their presence is unknown [3].

## Figures and Tables

**Table 1:** Sequences of primers used in qPCR measurements.

Subunit	Forward	Reverse
*α*2*δ*1	AGGCAGTTGAGATGGAGGAA	CCCTTTGCTCTCCACCATTA
*α*1s	AGGTCATGGACGTGGACGACTTGAG	CCAGGTTGCTCAGCGATGTCCAGTA
*β*	CCGGACCTTGCAGCTGGTCG	GGATTGGGTGGCGTGCTGCT
*γ*	GGCCGTGCTGAGTCCACACC	GGAATGGCCGCTGCTGAGA
MyoD	AGGCTCTGCTGCGCGACCA	CCAGGAGTGCCTACGGTGGTG
Myogenin	AGTGAATGCAACTCCCCACAGC	TGTGGCTTGTGGCAGCCCAG
18S	AATTGACGGAAGGGCACCAC	GTGCAGCCCCGGACATCTTAAG
HPRT1	TCAGTCAACGGGGGACATAAA	GGGGCTGTACTGCTTAACCAG
YWHAZ	AACAGCTTTCGATGAAGCCAT	TGGGTATCCGATGTCCACAAT

**Table 2:** Distribution of sorted cells by groups. Mean ± sem, *n* = 11.

*α*2*δ*1^+^/CD34^−^	*α*2*δ*1^+^/CD34^+^
21 ± 3.7%	32 ± 4.5%

*α*2*δ*1^−^/CD34^−^	*α*2*δ*1^−^/CD34^+^
44 ± 5.3%	3.0 ± 1.2%
